# Evaluation of lateral flow devices for rabies diagnosis in decomposed animal brain samples

**DOI:** 10.1186/s41182-025-00699-4

**Published:** 2025-02-25

**Authors:** Ryota Todoroki, Joely T. Ongtangco, Kazunori Kimitsuki, Nobuo Saito, Milagros R. Mananggit, Cornelio R. Velasco, Jaira D. Mauhay, Alyssa M. Garcia, Catalino S. Demetria, Yamada Kentaro, Akira Nishizono

**Affiliations:** 1 Department of Microbiology, Faculty of Medicine, Oita University, Yufu, Oita Japan; 2 Regional Animal Disease Diagnostic Laboratory, Department of Agriculture, Regional Field Office III, Tarlac, Tarlac Philippines; 3 Research Center for Global and Local Infectious Diseases, Oita University, Yufu, Oita Japan; 4 Kenya Research Station, Institute of Tropical Medicine, Nagasaki University, Nagasaki, Nagasaki Japan; 5 Research Institute for Tropical Medicine, Muntinlupa, Metro Manila Philippines; 6 Laboratory of Veterinary Public Health, Department of Veterinary Science, Faculty of Agriculture, University of Miyazaki, Miyazaki, Miyazaki Japan

**Keywords:** LFD, DFAT, Rabies, Decomposed sample, The Philippines

## Abstract

**Background:**

The Direct Fluorescent Antibody Test (DFAT), the standard rabies confirmatory test, is less sensitive when used with decomposed brain samples, a frequent issue in rabies-endemic regions. This study evaluates the diagnostic accuracy of the ADTEC lateral flow device (LFD) for rabies post-mortem diagnosis using decomposed brain samples.

**Methods:**

We used 34 animal heads submitted with a cold chain to an animal diagnostic laboratory located in central Philippines including 26 DFAT-positive and eight DFAT-negative samples. After defrosting the heads, the entire brain was extracted and left at room temperature to induce decomposition. The decomposition status was scored after 1 day, 3 days, and 4 days at room temperature. DFAT and LFD were performed using the brain samples at each timepoint to evaluate the diagnostic accuracy. The day of animal head submission to the laboratory was defined as day 0, and the DFAT results were used as the reference. Image analysis was performed to measure the intensity of the LFD-positive bands.

**Results:**

The decomposition scores dropped by day 3 and day 4, with all samples exhibiting signs of advanced decomposition. The sensitivity of DFAT was 96.2% (95% confidence interval 80.4–99.9) on day 1, but dropped to 61.5% (*P* < 0.01) by day 3 and further decreased to 38.5% (20.2–59.4) by day 4. In contrast, the sensitivities and specificities of LFD on day 1, day 3, and day 4 were consistently 100% (86.3–100) and 100% (63.1–100), respectively. Visual examination of the test band intensity on the LFD suggested that the intensity of the positive bands increased as decomposition progressed.

**Conclusions:**

ADTEC LFDs demonstrated consistently high sensitivity and specificity with decomposed brain samples observed up to day 4, making them a reliable screening tool for rabies post-mortem diagnosis in decomposed brain samples, particularly in resource-limited settings. Furthermore, LFD positive bands became more distinct as decomposition advanced.

**Supplementary Information:**

The online version contains supplementary material available at 10.1186/s41182-025-00699-4.

## Introduction

Rabies continues to be a public health issue in Asian and African countries, with domestic dogs being the primary source of human deaths [1]. Rapid and accurate diagnostic tests in suspected animals are essential for controlling ongoing transmission and raising awareness in the community [2, 3]. A lack of a confirmatory test hinders the precise determination of areas affected by rabies outbreaks, thereby contributing to the spread of the virus. Current confirmatory tests for rabies, such as direct fluorescent antibody test (DFAT), mainly require brain tissues [2, 3]. Rabies confirmatory tests only show positive results once the virus infects the central nervous system and the animal exhibits clinical symptoms of rabies. A limitation of DFAT is its reduced sensitivity when used on decomposed brain tissue [2, 4–6]. Brain specimens are often found in decomposed conditions, because the carcasses of stray dogs and wild animals are frequently found several days after death [7]. Furthermore, transporting brain samples from remote areas to an animal diagnostic laboratory is challenging due to their decomposability and the necessity of maintaining a cold chain [7, 8]. Our baseline survey of rabies diagnostic laboratories in the Philippines revealed that 1–5% of samples were unfit for DFAT due to decomposition, while another study found that 9.3% of samples were transported at room temperature without cold chain preservation [9, 10].

Several studies showed that the diagnostic accuracy of DFAT diminishes when the samples used were decomposed [4, 5, 11, 12]. To ensure optimal accuracy, DFAT is recommended to be performed within 24–48 h, particularly in environments, where temperatures reach approximately 30 °C [4, 6, 13, 14]. McElhinney et al. [5] investigated the effects of decomposition on rabies detection using DFAT, the Rabies Tissue Culture Inoculation Test (RTCIT), and RT-PCR on brain tissue from infected mouse carcasses. The study revealed that as both temperature increased and decomposition time lengthened, the detection sensitivity of all methods decreased. The detection periods were also shortened in the following order: RTCIT, DFAT, and RT-PCR, with RT-PCR being the most robust, sustaining its ability to detect the virus for the longest period [5]. Albas et al. examined the detectability of rabies in the decomposing brains of ten canine carcasses that had died from rabies, by storing them at room temperature for various durations and testing them using the DFAT and mouse inoculation test (MIT) [6]. MIT detection was only feasible up to 48 h, while DFAT failed to detect rabies in 2 out of 10 samples by 96 h and in 8 out of 10 samples by 120 h. Several reports indicated that RT-PCR showed positive results in cases, where animal carcasses left at room temperature for prolonged durations were unfit for DFAT due to their decomposed state [4, 7, 14–16]. Although molecular methods have demonstrated sufficient diagnostic accuracy with decomposed brain samples, many frontline laboratories in developing countries, especially in remote areas, lack the necessary facilities to perform these tests. In addition, contamination issues during necropsy procedure can result in false positives in molecular assays [17].

Lateral flow devices (LFDs) have been developed for rabies rapid tests, offering advantages, such as speed, ease of use, and no need for additional equipment. Although various types of LFDs have been developed and marketed, their insufficient sensitivity has been a concern [18]. However, the ADTEC LFD, developed using colloidal-gold-labeled monoclonal antibodies against the N protein of the rabies virus, has shown a sufficiently high diagnostic accuracy [9, 10, 19, 20]. A multicenter study in the Philippines confirmed its high diagnostic accuracy, with a sensitivity of 96.3% [95% confidence interval (CI), 88–98%] and a specificity of 99.7% (95% CI, 98.4–100%) [9]. Although systematic evaluations of LFD sensitivity for decomposed samples are scarce, one study reports that rabies can be detected over an extended period using LFD [21]. If decomposed samples can be accurately tested using LFD, it would enable simple and accessible testing in any location when laboratories receive decomposed samples. This study aimed to evaluate the diagnostic accuracy of LFD on decomposed brain samples.

## Methods

### Ethics statement

The institutional animal care and use committee at the Research Institute of Tropical Medicine (RITM) waived the ethical approval, since this study only collected brain samples from carcasses or animal heads submitted to routine surveillance. For biosafety clearance, our research protocol was approved by the biosafety committee of RITM (no.190116). This biological material reflects the conditions relevant to rabies diagnosis, considering animal species, age, sex, household management, and the time from death to arrival at the diagnostic laboratory. This approach allows for an evaluation closely aligned with the actual circumstances of rabies diagnosis without causing additional harm to animals. In this study, we used only animal information collected for routine surveillance after excluding any individual identifiable information.

### Study site

This study was conducted at the regional animal diagnostic laboratory under Philippine Department of Agriculture, located in Tarlac province, Region 3, the Philippines. The laboratory accepts samples for animal diagnostic tests in the region. The population in the region is 12 million (2020 census), making it the second most densely populated area in the Philippines after Metro Manila. The region has the highest incidence of rabies in the country, with 54 human rabies cases and 368 rabid animals reported in 2023 (unofficial report by the Philippine Department of Health and the Department of Agriculture, Regional Office).

### Samples

The laboratory provides free animal rabies testing by DFAT to local government agencies and residents from the northern provinces of the region. Animals suspected of rabies are usually found dead or are euthanized by local authorities. Subsequently, an animal head is removed and transported to the laboratory for rabies testing under frozen conditions. In the laboratory, a straw sampling method is routinely used to collect brain tissues for DFAT [10]. A plastic straw is inserted into foramen magnum of animal heads to obtain small portions of brain tissue. In this study, we used animal heads that had been processed with the straw sampling method and tested with DFAT on the day the samples were submitted. The DFAT results were denoted as “day 0”. Using DFAT results from day 0 as the reference, we evaluated sensitivity and specificity of both LFD and DFAT following decomposition. This study utilized 26 DFAT-positive and eight DFAT-negative animals collected randomly between March and June 2023. After the routine procedures of the straw sampling method and DFAT, the whole brains were extracted following the opening of the skull and then stored in a −80 °C freezer.

### Decomposition experiment

At the beginning of each decomposition experiment, 8–12 brain samples were left at room temperature inside a biosafety cabinet. To prevent cross-contamination during the decomposition experiments, we ensured that sampling tools were replaced, and gloves were changed between handling each brain sample. In addition, each sample was placed in a separate petri dish to further minimize the risk of cross-contamination. Portions of the medulla were then collected after 24 h (day 1), 72 h (day 3), and 96 h (day 4) and were subsequently used for DFAT and LFD. The positive or negative results for each animal were determined based on the results of the DFAT on day 0 and were compared with the test results for each sample collected on indicated days. DFAT and LFD were performed immediately after the sample collection. To assess the decomposition status of brain samples, we used our original scoring system developed in this study. The scoring system was modified from that used by the Central Animal Laboratory of the Bureau of Animal Industry under the Philippine Department of Agriculture. The scoring system included shape, appearance, consistency, and color, each rated on a three-point scale. The total score was used to assess the degree of decomposition, with the highest level of decomposition scoring 4 points and 12 points for no decomposition (Additional file 1: Table S1). Next, the temperature and humidity of the atmospheric air were measured at 9:00, 13:00, and 17:00 each day. In this building, the air conditioning operates during the daytime and is turned off at night. The air conditioning is set to turn on at 9:00 in the morning.

### DFAT and LFD

The methods for DFAT and LFD were performed as described in previous studies [10, 19]. Briefly, an impression of one portion of the medulla was stained with fluorescein isothiocyanate-conjugated anti-rabies monoclonal antibody (Fujirebio, Malvern, PA, USA). The stained samples were examined under a fluorescence microscope by two independent examiners. Subsequently, the same portion of the medulla was used for LFD examinations. We used ADTEC LFD (ADTEC Co., Ltd., Oita, Japan) for this study, following the company’s instructions. Currently, ADTEC LFD is available in the Philippines only for research purposes, costing approximately 1,000 Philippine pesos (about US$17) per test, and is recommended to be stored under refrigerated conditions prior to use. An approximately 5-mm diameter portion of the medulla was placed in a tube and homogenized with assay buffer using the pestle provided in the kit. The homogenized sample was then added to the kit, and the results were evaluated after 15 min. The LFD results were interpreted by a different examiner from the DFAT examiner, with both tests conducted in a blind manner to ensure independent evaluation.

### Data analysis

The sensitivity and specificity of the DFAT and LFD on day 1, day 3, and day 4, along with 95% confidence intervals, were determined with the DFAT results on day 0 as the reference test. The sensitivities of DFAT and LFD on day 1, day 3 and day 4 were presented. We analyzed the band intensities of test and control on LFD strips using the open-source software ImageJ (https://imagej.net/ij/index.html) [22]. After capturing images of the LFDs by smartphone, we converted these pictures to 8-bit grayscale to ensure uniformity in the analysis. The software converted each detected band into a peak that reflected the pixel count, and the area under the curve (AUC) represented the band’s intensity. We calculated the AUC ratios of the test band to the control band (T/C ratio) [23, 24]. We compared the T/C ratios on day 1, day 3, and day 4 using Wilcoxon signed-rank test. Samples with no positive bands detected, or where we failed to obtain images (ID = 110, 207), were excluded from the analysis. The study adhered to the guidelines outlined in the STARD (Strengthening the Reporting of Observational Studies in Epidemiology) statement (Additional file 2: Table S2). We conducted this study as an initial pilot trial, without performing a sample size calculation for diagnostic accuracy. Instead, the study was designed around the available study duration and the number of brain samples that could be stored in the biosafety facility each time, which had a maximum capacity of 12.

## Results

During the observation period, the temperature in the laboratory room ranged from 24.4 to 28.2 °C before the air conditioning started each morning, stabilizing between 22.0 and 25.0 °C during the day, with humidity levels ranging from 60 to 84%. A total of 34 animals were included in this study, with 26 (76.5%) DFAT-positive and 8 (23.5%) DFAT-negative samples. Of the 34 animals, 31 (91.2%) were dogs with 26 DFAT positive, and 3 (8.8%) were cats with all DFAT-negative (Table 1). Thirteen animals (38.2%) were under 12 months of age, and 18 (52.9%) were female (Table 1).

**Table 1:** Characteristics of 34 animal samples by DFAT results on day 0

		Total, N (%)	DFAT Negative, N (%)	DFAT Positive, N (%)
Total		34	8 (23.5)	26 (76.5)
Species	Dog	31 (91.2)	5 (62.5)	26 (100)
	Cat	3 (8.8)	3 (37.5)	0 (0)
Age	<12 months	13 (38.2)	5 (62.5)	8 (30.8)
	1–2 years	7 (20.6)	1 (12.5)	6 (23.1)
	2–5 years	8 (23.5)	1 (12.5)	7 (26.9)
	>5 years	2 (5.9)	0 (0)	2 (7.7)
	Unknown	4 (11.8)	1 (12.5)	3 (11.5)
Sex	Female	18 (52.9)	4 (50.0)	14 (53.8)
	Male	13 (38.2)	3 (37.5)	10 (38.5)
	Unknown	3 (8.8)	1 (12.5)	2 (7.7)

*DFAT* direct fluorescent antibody test

**Fig. 1:**
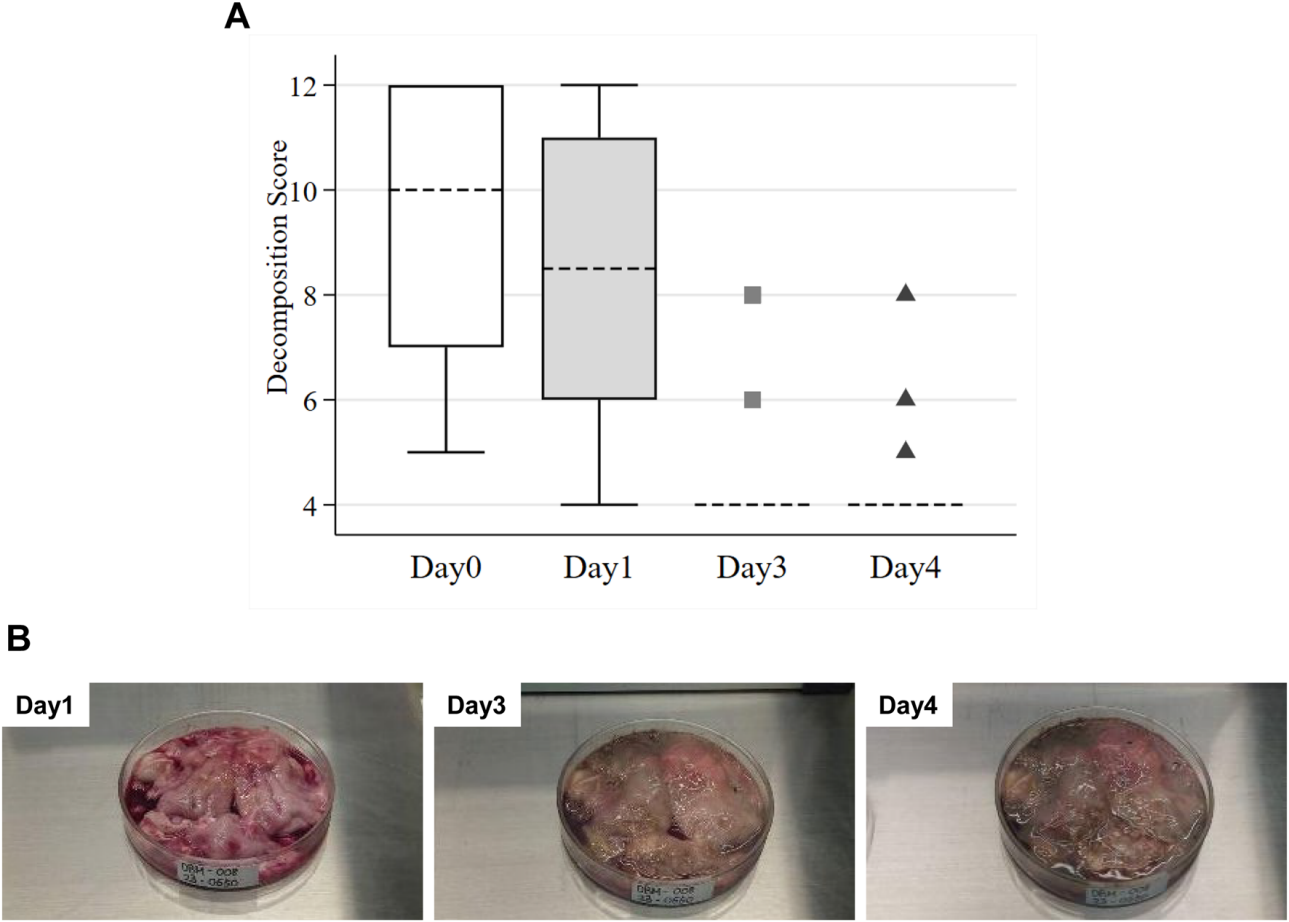
Decomposition scores and representative brain images on day 1, day 3, and day 4, **A** Median decomposition scores with interquartile range (IQR) on days 1, 3, and 4 (A). The dashed line represents the median, the box indicates IQR and the whiskers show the full range of the data. The gray squares ( ) and black triangles (▲) represent outliers. **B** Representative case (ID: DBM-008) of brain decomposition on days 1, 3, and 4. Decomposition scores were 8 on day 1, 4 on day 3, and 4 on day 4, respectively

Notable decomposition in the brain was observed on days 3 and 4 compared to day 1 (Fig. 1 and Additional file 3: Table S3). For decomposition scores, the median (range) values were 10 (5–12) on day 0, 9 (4–12) on day 1, 4 (4–8) on day 3, and 4 (4–8) on day 4, indicating a notable decrease in decomposition scores by day 3 and day 4, with all samples exhibiting signs of severe decomposition (Fig. 1 and Additional file 3: Table S3). With the DFAT results on day 0 as the reference, the sensitivity of DFAT on day 1 was 96.2% (95% CI 80.4–99.9), which dropped to 61.5% (40.6–79.8) on day 3, and further decreased to 38.5% (20.2–59.4) on day 4 (Table 2).

**Table 2:** Diagnostic accuracy of DFAT and LFD on day 1, day 3, and day 4 compared with DFAT on day 0 as the reference

		DFAT Day 0 results		Sensitivity (95% CI)	Specificity (95% CI)
		Positive	Negative		
DFAT day 1	Positive	25	0	96.2 (80.4–99.9)	100 (63.1–100)
	Negative	1	8		
DFAT day 3	Positive	16	0	61.5 (40.6–79.8)	100 (63.1–100)
	Negative	10	8		
DFAT day 4	Positive	10	0	38.5 (20.2–59.4)	100 (63.1–100)
	Negative	13	8		
LFD day 1	Positive	26	0	100 (86.3–100)	100 (63.1–100)
	Negative	0	8		
LFD day 3	Positive	26	0	100 (86.3–100)	100 (63.1–100)
	Negative	0	8		
LFD day 4	Positive	26	0	100 (86.3–100)	100 (63.1–100)
	Negative	0	8		

*DFAT* Direct Fluorescent Antibody Test, *LFD* Lateral Flow Device, *95% CI* 95% Confidence Interval

**Fig. 2:**
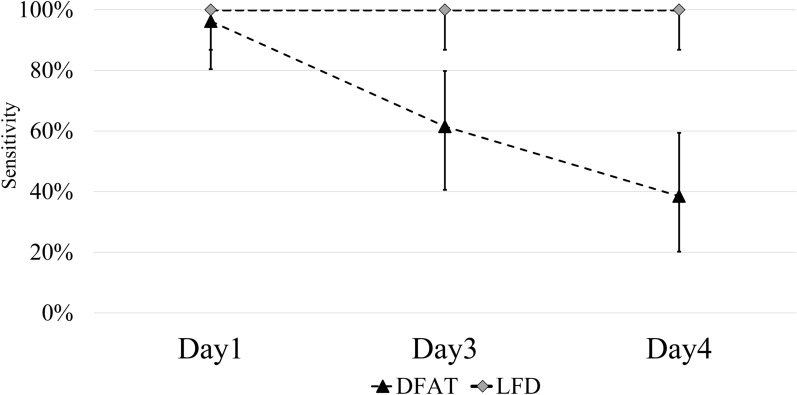
Comparative sensitivity of DFAT and LFD on day 1, day 3, and day 4. DFAT, Direct fluorescent antibody test; LFD, Lateral flow device. The *P* values were calculated using a proportion test to compare the sensitivity of DFAT and LFD on day 1, day 3, and day 4

**Fig. 3:**
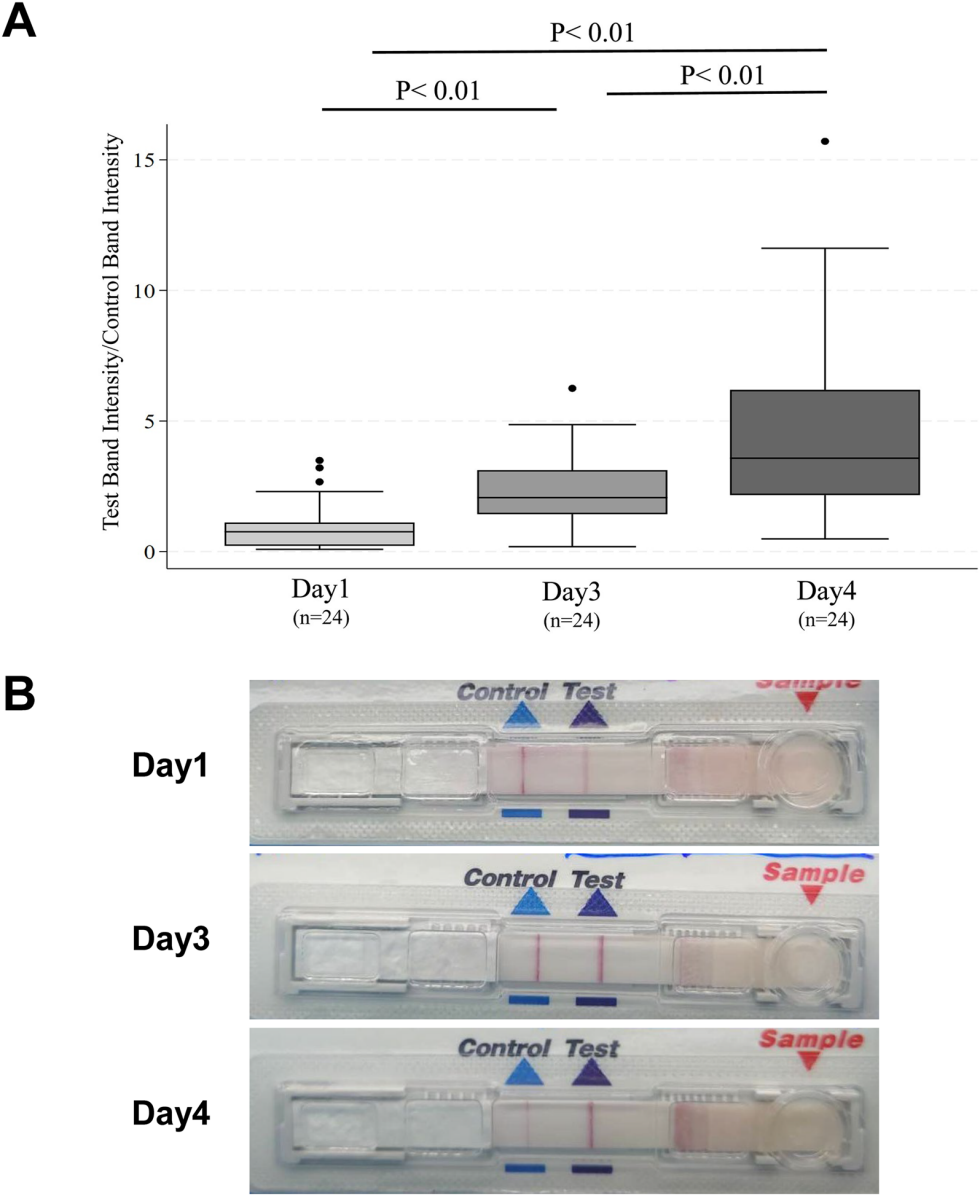
Visual comparison of LFD test band/control (T/C) band intensity ratio on day 1, day 3, and day 4. **A** T/C Band intensity ratios on days 1, 3, and 4. The dashed line represents the median, the box indicates the interquartile range (IQR) and the dots show the full range of the data. *P* value was calculated by Wilcoxon signed-rank test. Samples with no positive bands detected or for which images were unavailable (ID = 110, 207) were excluded from the analysis. The band intensity is represented by the area under the curve (AUC), calculated using the software ImageJ [https://imagej.net/ij/index.html]. **B** Representative case (ID: DBM-008) of LFD T/C band intensity on days 1, 3, and 4

While the sensitivity of DFAT was nearly the same as that of LFD on day 1, it became lower compared to LFD on day 3 and day 4 (Fig. 2). In contrast, the sensitivities and specificities of LFD on day 1, day 3, and day 4 were 100% (86.3–100) and 100% (63.1–100), respectively, with no false positives or false negatives observed, even after decomposition. In addition, visual examination of the LFD result images suggested that the intensity of the positive bands increased as decomposition progressed, with the median T/C ratios rising from 0.76 (interquartile range 0.22–1.11) on day 1 to 2.06 (1.43–3.12) on day 3, and 3.57 (2.16–6.19) on day 4 (Fig. 3 and Additional file 3: Table S3). Compared to day 1, the T/C ratios on day 3 (*P* < 0.01) and day 4 (*P* < 0.01) were significantly higher, with day 4 showing a significantly higher T/C ratio than on day 3 (Fig. 3). We selected four brain samples that remained positive on day 4 and tested them again on day 7 and day 9 using only LFD, with all samples still testing positive.

## Discussion

This study, involving 34 brain samples kept at room temperature to induce decomposition, found that the sensitivity of DFAT for rabies post-mortem diagnosis decreased as decomposition progressed. In contrast, LFD maintained a high diagnostic sensitivity and specificity regardless of the level of decomposition. In addition, the intensity of the positive bands on LFD tended to increase as decomposition progressed. These results suggest that decomposed samples are not suitable for DFAT; however, LFD can serve as a screening tool for decomposed samples particularly where nucleic acid amplification tests are not feasible. LFD may become increasingly effective at detecting antigens as decomposition progresses.

### LFD results and the decomposition progress

When performing DFAT in this study, we observed increased non-specific fluorescence and reductions in the typical signs of RABV antigen fluorescence (sparkling apple-green fluorescence) as decomposition progressed. This phenomenon is likely due to non-specific fluorescence caused by secondary bacterial contamination and the degradation of RABV antigens. These factors significantly complicated the interpretation of DFAT. However, these detection issues related to decomposition were not observed with LFD, which consistently yielded clear positive results. Notably, in our observations, the majority of positive bands on the LFD were stronger in decomposed stages compared to earlier stages. These results may suggest that DFAT and LFD are detecting different antigenic components. Although not clearly proven, DFAT primarily visualizes cytoplasmic inclusion bodies in nerve cells specifically Negri bodies, which contain the viral N and P proteins [25]. We hypothesized that decomposition likely disrupts the structure of Negri bodies, hindering their detection sensitivity by DFAT, while the antigens remain intact and are still detected by LFD. The increased intensity observed in LFD post-decomposition can possibly be explained by the disintegration of Negri bodies, leading to the release of antigens that can be detected by the antibodies of LFD.

There is also a possibility that the difference in antibodies used in DFAT and LFD might influence this phenomenon. Further studies are needed to clarify the reduction in Negri bodies and changes in antigen levels during decomposition. Our findings remain unproven and are limited by the lack of precise antigen quantification methods like ELISA. Furthermore, this study had a limitation in inconsistent brain tissue sampling, which may have influenced test band intensity.

### Detection duration

Although we demonstrated the high diagnostic accuracy of LFD in advanced stages of decomposition, this study was unable to determine the maximum detection duration. Due to our laboratory capacity limitations, further analysis beyond 4 days was not feasible; however, we were able to test only four samples on day 7 and day 9, with all samples still testing positive. For DFAT, as observed in this study and supported by others, sensitivity decreases after 48 h of decomposition [6, 26]. Nucleic acid amplification tests have been reported to detect rabies in samples left at room temperature or buried for long periods of time, with one study confirming detection on day 120 post-mortem [12, 27]. However, no study has thoroughly investigated how long LFD remains positive after decomposition. In a study by Tessy et al. [21], where the brains of eight rabid dogs were allowed to decompose, all samples remained LFD-positive up to day 24. By day 40, 87.5% of the samples were still positive; 50% were positive by day 50, and 25% remained positive by day 60. Thus, LFD has the potential to detect rabies antigens for extended periods, even in decomposed samples.

## Limitations

This study has several limitations. First, the sample size was relatively small for the assessment of diagnostic accuracy. This study was designed as an initial pilot trial and faced many challenges in processing a large number of samples for decomposition experiments. However, despite the small sample size, we observed a clear difference in sensitivity between LFD and DFAT when testing decomposed brain samples. Second, we were unable to present RT-PCR results in this study due to a contamination issue. We performed RT-PCR on frozen brain samples obtained in this study but were unable resolve the contamination issue despite taking extensive precautions, such as frequently replacing equipment and gloves. We believe this issue was likely caused by the environmental conditions of the dissection room, which we routinely use for processing a large number of infected animal samples, such as rabies-infected brain samples, potentially leading to contamination. Due to these issues, we recognized the need to recollect samples suspected of cross-contamination. However, the nature of the experiment, involving daily sampling of decomposed brain samples, made this challenging. As a result, we deemed these data inappropriate and excluded them from the findings of this study. In addition, since we tested only up to day 4 samples, we were unable to identify the maximum duration for LFD detection, which requires further assessment. Further research is necessary to understand the association between the decomposition status and antigen levels using quantification methods, such as ELISA.

In conclusion, when using decomposed brain samples, LFD demonstrated higher sensitivity compared to DFAT. The intensity of the LFD bands increased as decomposition progressed. These findings support the prioritization of LFD as a screening test for rabies in decomposed samples in resource-limited settings.

## Supplementary Information

Additional file 1.

Additional file 2.

Additional file 3.

Additional file 4.

## Data Availability

The data set presented in this study is available on the Oita University Repository (http://hdl.handle.net/10559/0002013324).
