# Supplementary material for: Evaluation of lateral flow devices for rabies diagnosis in decomposed animal brain samples
Source: Trop Med Health. 2025 Feb 25;53:30. doi: 10.1186/s41182-025-00699-4 (PMC11853130; doi:10.1186/s41182-025-00699-4)
Supplement: Supplementary file 1 — Additional file 1. [file 41182_2025_699_MOESM1_ESM.pdf]

Supporting information

"Evaluation of Lateral Flow Devices for Rabies Diagnosis in Decomposed Animal Brain Samples"

S1 Table. Decomposition score sheet for brain specimens: Evaluation criteria for rabies testing.

	1	2	3	Score
**Shape (1~3)**	***Indistinguishable Brain Structure:*** Severe damage or decomposition makes specific parts unidentifiable.	***Partially Distinguishable Brain Structure:*** Some areas identifiable; others obscured, damaged, or decomposed.	***Distinguishable Brain Structure*:** Mostly intact with recognizable parts (e.g., cerebellum, hippocampus, brainstem).	____
**Appearance (1~3)**	***Disrupted or Damaged:*** Tissue appears disrupted with areas of liquefaction or necrosis; pia mater obscured or missing.	***Partially Intact:*** Tissue shows damage with areas of preservation; pia mater may be partially visible.	***Intact and Healthy:*** Tissue appears robust; pia mater completely visible with no signs of damage.	____
**Consistency (1~3)**	***Soft:*** Tissue is soft, easily disintegrated, indicating severe degeneration or autolysis.	***Moderately Firm:*** Tissue has some firmness, disintegrates with moderate force, suggesting mild degeneration or autolysis.	***Hard:*** Tissue is firm, resilient, and indicative of healthy or fresh tissue.	____
**Color (1~3)**	***Dark/Discolored:*** Tissue is dark, discolored due to hemorrhage, necrosis, or decomposition.	***Pale:*** Tissue appears paler than typical, suggesting edema or mild decomposition.	***Normal Color:*** Tissue is pinkish-grey, indicating healthy or fresh tissue.	____
			**Total Score** (Poor 4-6, Good 7-9, Well-Preserved 9-12)	___
