# Supplementary material for: Evaluation of lateral flow devices for rabies diagnosis in decomposed animal brain samples
Source: Trop Med Health. 2025 Feb 25;53:30. doi: 10.1186/s41182-025-00699-4 (PMC11853130; doi:10.1186/s41182-025-00699-4)

S2 Table. STARD (Standards for Reporting Diagnostic accuracy studies) checklist

Section & Topic	No	Item	Reported on page #
**TITLE OR ABSTRACT**			
	**1**	Identification as a study of diagnostic accuracy using at least one measure of accuracy (such as sensitivity, specificity, predictive values, or AUC)	Page 1, Title
**ABSTRACT**			
	**2**	Structured summary of study design, methods, results, and conclusions (for specific guidance, see STARD for Abstracts)	Page 3-4 abstract
**INTRODUCTION**			
	**3**	Scientific and clinical background, including the intended use and clinical role of the index test	Page 5-7
	**4**	Study objectives and hypotheses	Page 7
**METHODS**			
Study design	**5**	Whether data collection was planned before the index test and reference standard were performed (prospective study) or after (retrospective study)	Page 9
Participants	**6**	Eligibility criteria	Page 10-12
	**7**	On what basis potentially eligible participants were identified (such as symptoms, results from previous tests, inclusion in registry)	Page 8-9
	**8**	Where and when potentially eligible participants were identified (setting, location and dates)	Page 8-9
	**9**	Whether participants formed a consecutive, random or convenience series	Page 8-9
Test methods	**10a**	Index test, in sufficient detail to allow replication	Page 9-10
	**10b**	Reference standard, in sufficient detail to allow replication	Page 10-11
	**11**	Rationale for choosing the reference standard (if alternatives exist)	Page 5
	**12a**	Definition of and rationale for test positivity cut-offs or result categories of the index test, distinguishing pre-specified from exploratory	Reference 21
	**12b**	Definition of and rationale for test positivity cut-offs or result categories of the reference standard, distinguishing pre-specified from exploratory	Reference 10, 24
	**13a**	Whether clinical information and reference standard results were available to the performers/readers of the index test	Page 11
	**13b**	Whether clinical information and index test results were available to the assessors of the reference standard	Page 11
Analysis	**14**	Methods for estimating or comparing measures of diagnostic accuracy	Page 11
	**15**	How indeterminate index test or reference standard results were handled	Page 11
	**16**	How missing data on the index test and reference standard were handled	NA
	**17**	Any analyses of variability in diagnostic accuracy, distinguishing pre-specified from exploratory	Page 11-12
	**18**	Intended sample size and how it was determined	Page 12
**RESULTS**			
Participants	**19**	Flow of participants, using a diagram	NA
	**20**	Baseline demographic and clinical characteristics of participants	Page 12-13, table1
	**21a**	Distribution of severity of disease in those with the target condition	NA
	**21b**	Distribution of alternative diagnoses in those without the target condition	NA
	**22**	Time interval and any clinical interventions between index test and reference standard	NA
Test results	**23**	Cross tabulation of the index test results (or their distribution) by the results of the reference standard	Page 13, Table2
	**24**	Estimates of diagnostic accuracy and their precision (such as 95% confidence intervals)	Page 13, Table2
	**25**	Any adverse events from performing the index test or the reference standard	Page 13, Table2
**DISCUSSION**			
	**26**	Study limitations, including sources of potential bias, statistical uncertainty, and generalisability	Page 16-17
	**27**	Implications for practice, including the intended use and clinical role of the index test	Page 14 and 17

OTHER INFORMATION			
	**28**	Registration number and name of registry	Page 8
	**29**	Where the full study protocol can be accessed	NA
	**30**	Sources of funding and other support; role of funders	Page 18

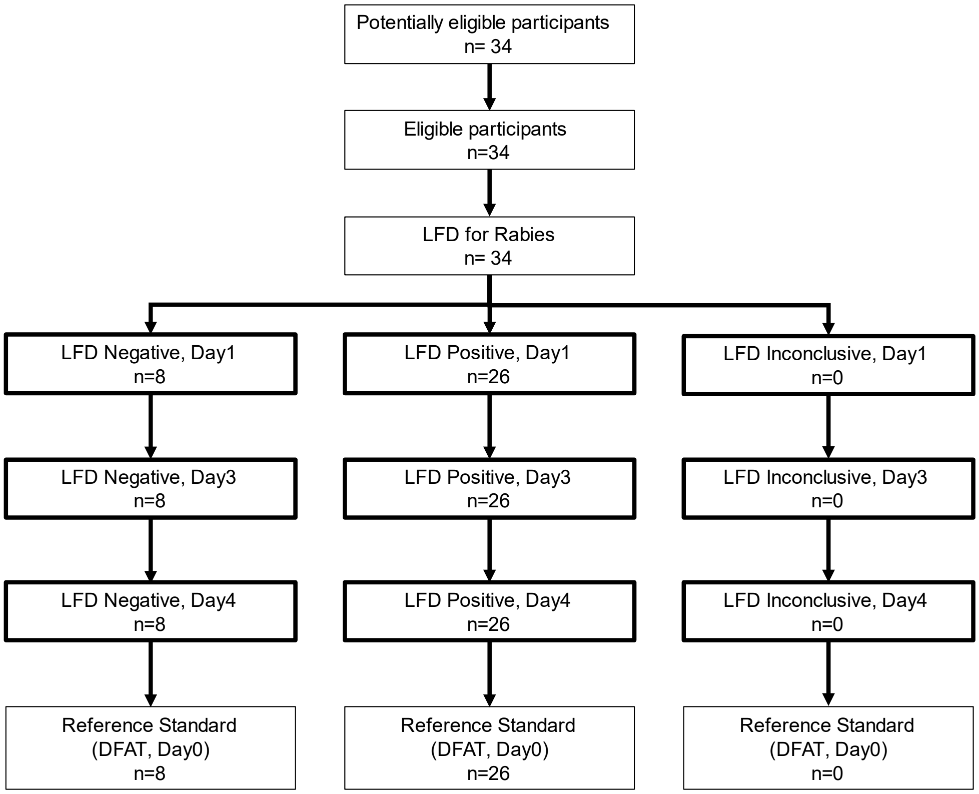

Supplement: Supplementary file 2 — Additional file 2. [file 41182_2025_699_MOESM2_ESM.pdf]
