# Supplementary material for: Evaluation of lateral flow devices for rabies diagnosis in decomposed animal brain samples
Source: Trop Med Health. 2025 Feb 25;53:30. doi: 10.1186/s41182-025-00699-4 (PMC11853130; doi:10.1186/s41182-025-00699-4)
Supplement: Supplementary file 3 — Additional file 3. [file 41182_2025_699_MOESM3_ESM.pdf]

Additional file 3 Table S3.

Images showing decomposition status of brain samples and LFD test band.

<table>
<tr><th>ID</th><th></th><th>Day1</th><th>Day3</th><th>Day4</th></tr>
<tr><td rowspan="3">001</td><td>Brain</td><td>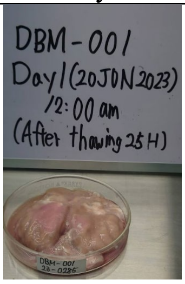</td><td>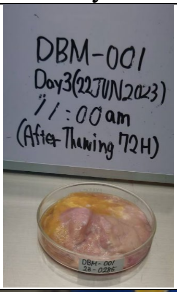</td><td>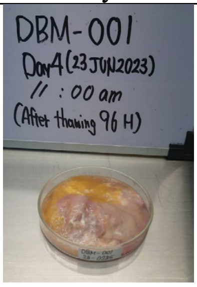</td></tr>
<tr><td>LFD
(Test band intensity)</td><td>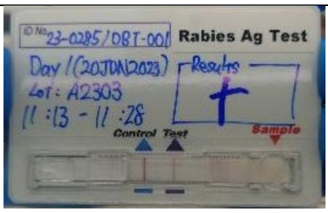
(966.355)</td><td>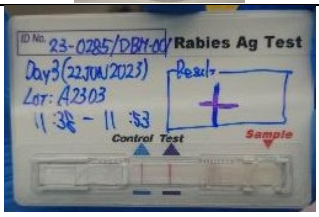
(2172.255)</td><td>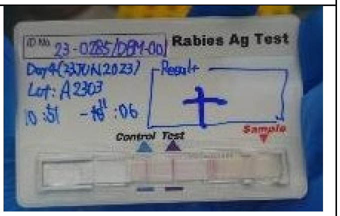
(1738.305)</td></tr>
<tr><td>DFAT</td><td>Positive</td><td>Positive</td><td>Positive</td></tr>
<tr><td rowspan="3">002</td><td>Brain</td><td>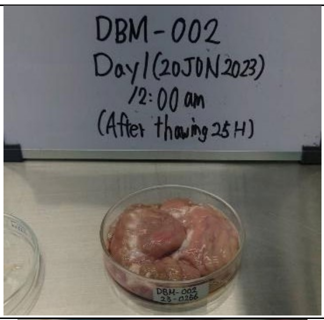</td><td>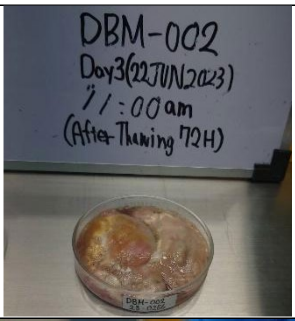</td><td>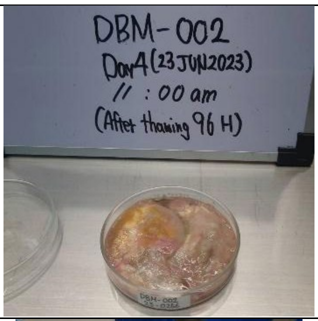</td></tr>
<tr><td>LFD
(Test band intensity)</td><td>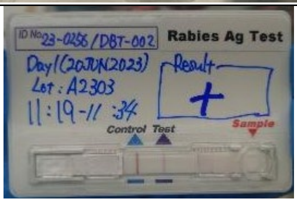
(508.749)</td><td>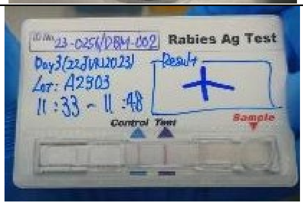
(2042.255)</td><td>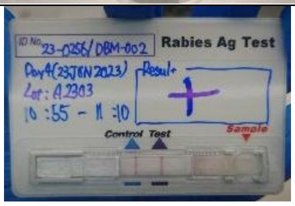
(1434.891)</td></tr>
<tr><td>DFAT</td><td>Positive</td><td>Negative</td><td>Negative</td></tr>
</table>

003	Brain	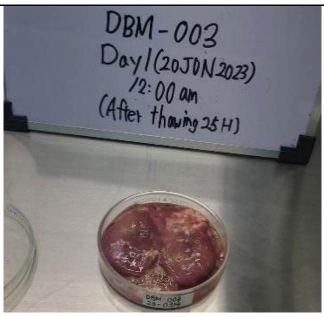	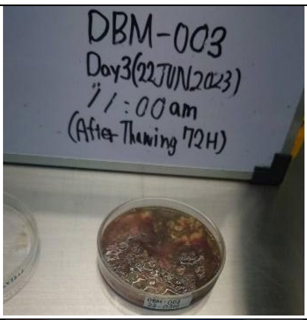	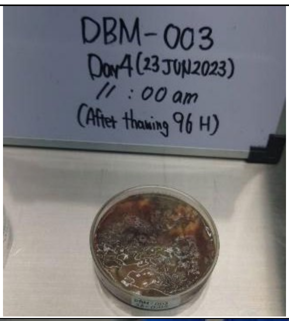
	LFD (Test band intensity)	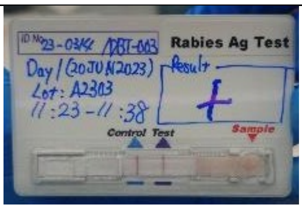 (798.234)	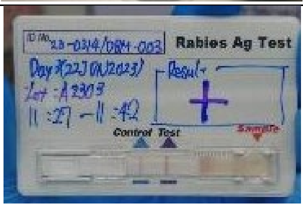 (1174.184)	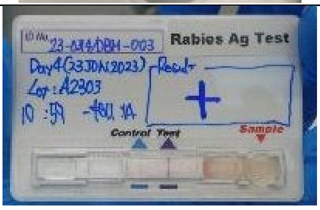 (906.234)
	DFAT	Positive	Negative	Negative
004	Brain	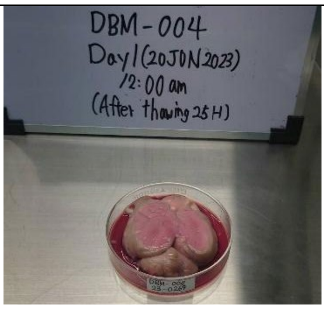	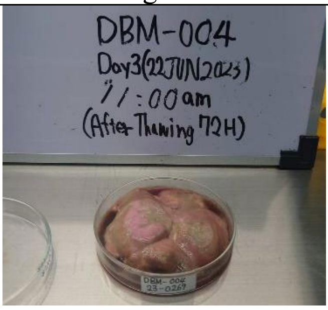	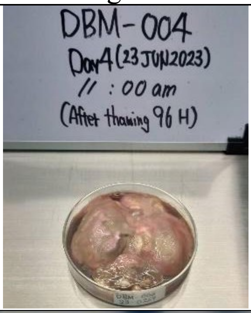
	LFD (Test band intensity)	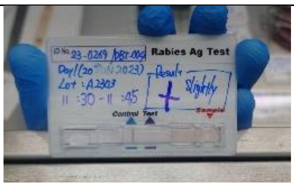 (120.364)	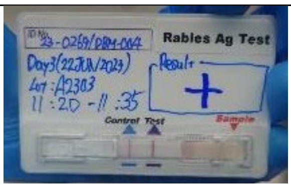 (2062.719)	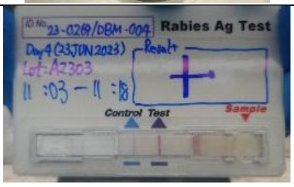 (2067.376)
	DFAT	Positive	Positive	Negative
005	Brain	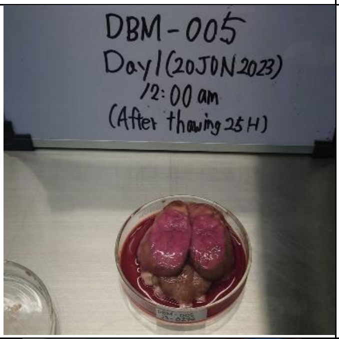	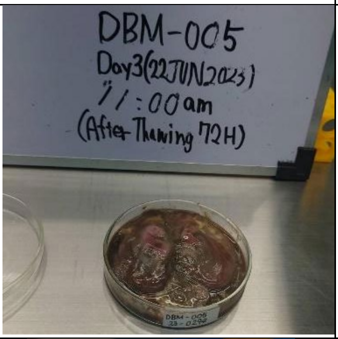	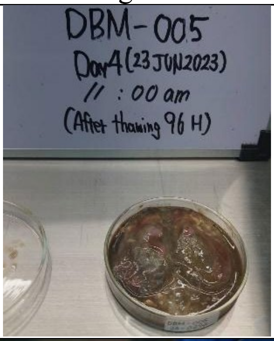
	LFD (Test band intensity)	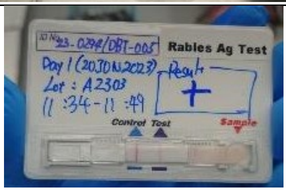 (210.85)	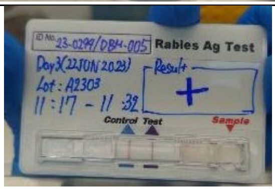 (2308.3)	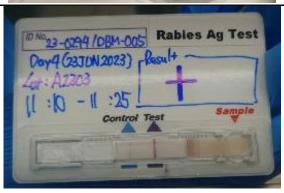 (2009)
	DFAT	Positive	Negative	Negative

006	Brain	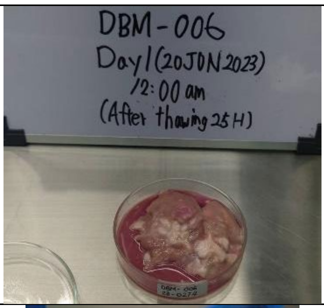	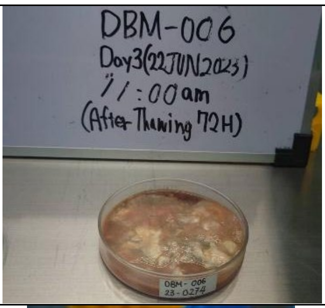	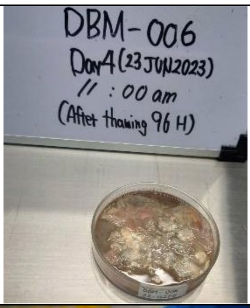
	LFD<br>(Test band intensity)	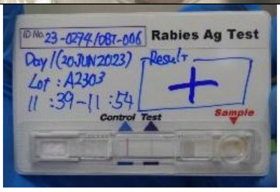<br>(210.849)	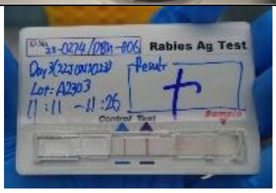<br>(2308.255)	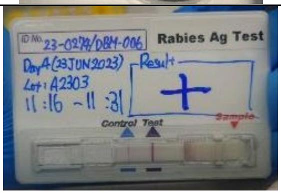<br>(2009.012)
	DFAT	Positive	Negative	Negative
007	Brain	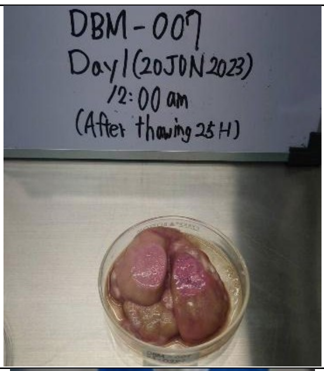	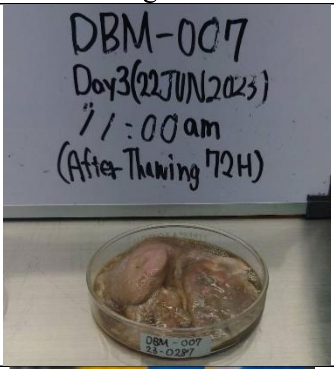	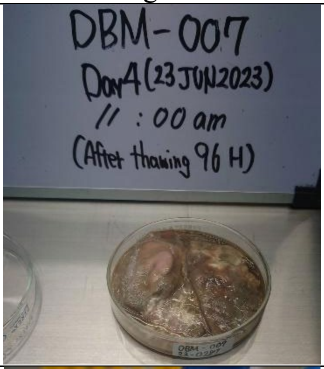
	LFD<br>(Test band intensity)	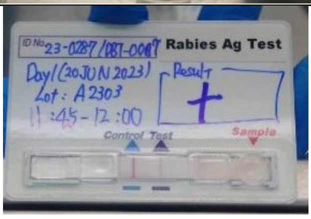<br>(216.435)	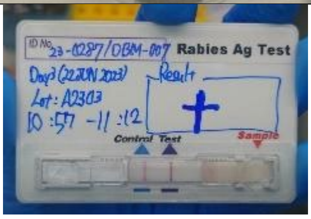<br>(1083.991)	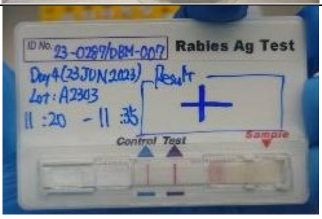<br>(2047.205)
	DFAT	Positive	Negative	Negative
008	Brain	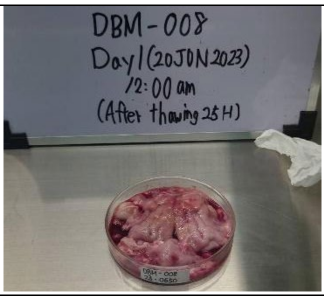	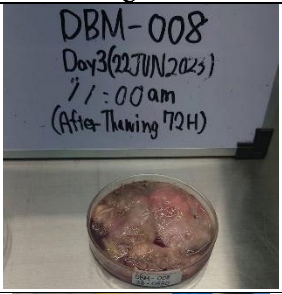	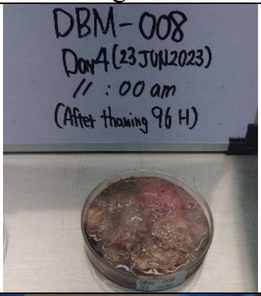
	LFD<br>(Test band intensity)	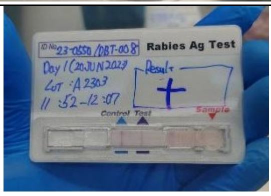<br>(585.87)	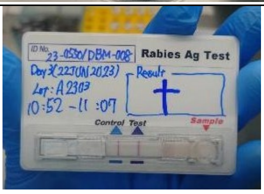<br>(1487.598)	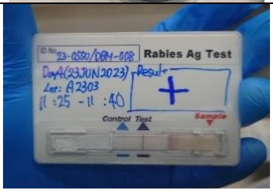<br>(1275.305)
	DFAT	Positive	Positive	Negative

009	Brain	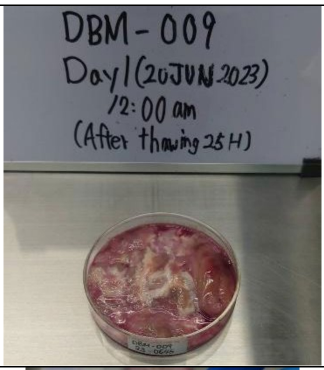	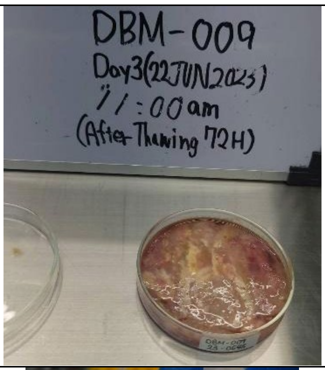	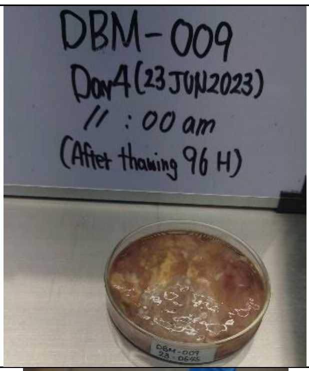
	LFD<br>(Test band intensity)	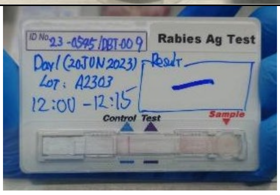<br>(0)	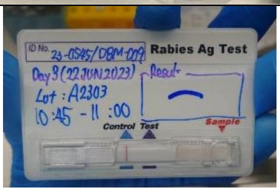<br>(0)	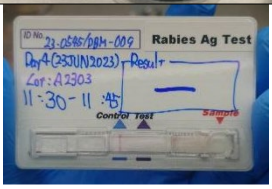<br>(0)
	DFAT	Negative	Negative	Negative
010	Brain	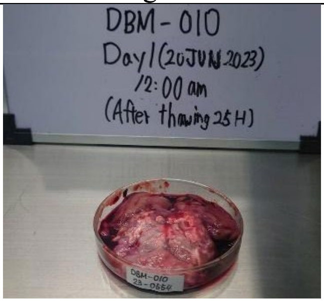	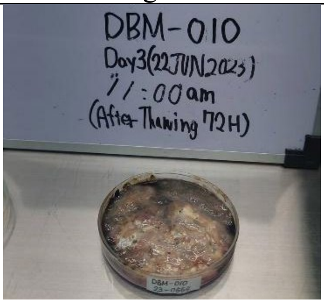	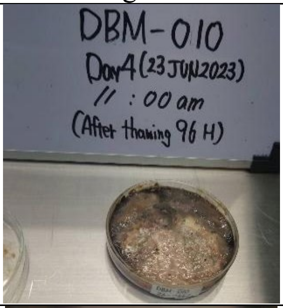
	LFD<br>(Test band intensity)	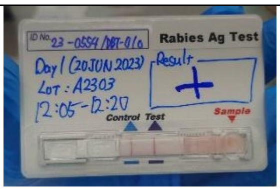<br>(1521.426)	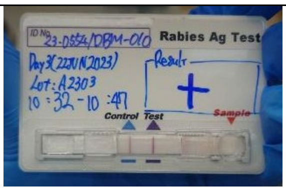<br>(2223.255)	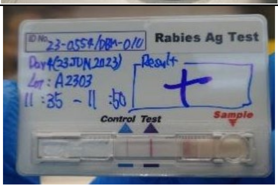<br>(2313.376)
	DFAT	Positive	Negative	Negative
011	Brain	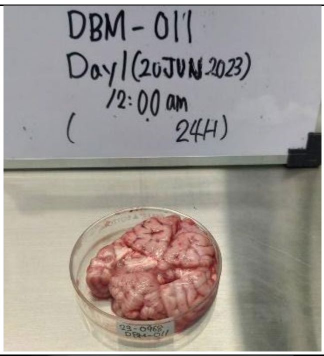	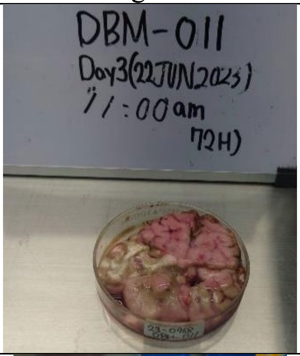	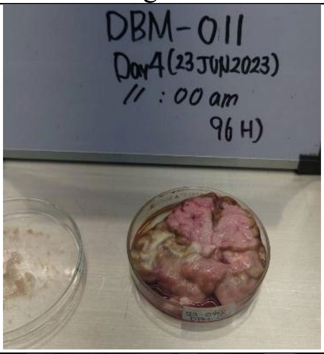
	LFD<br>(Test band intensity)	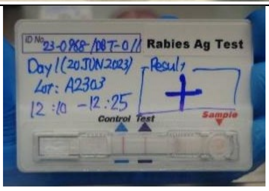<br>(680.577)	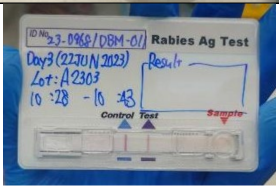<br>(1148.77)	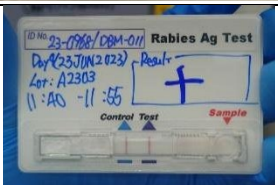<br>(1582.305)

	DFAT	Positive	Positive	Positive
102	Brain	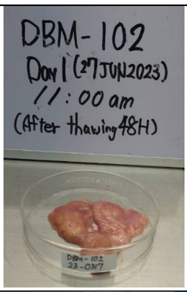	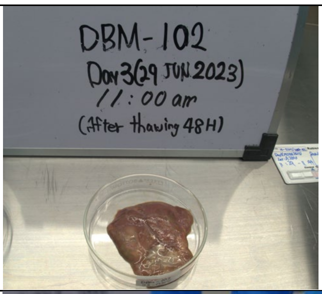	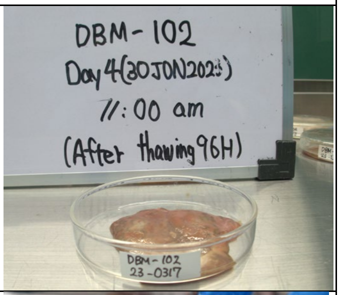
	LFD<br>(Test band intensity)	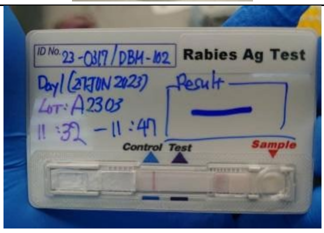<br>(0)	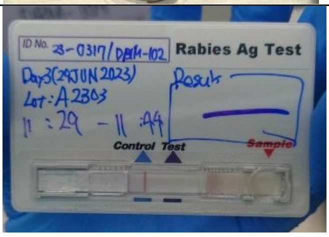<br>(0)	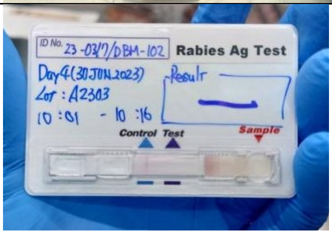<br>(0)
	DFAT	Negative	Negative	Negative
103	Brain	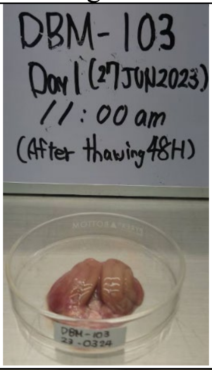	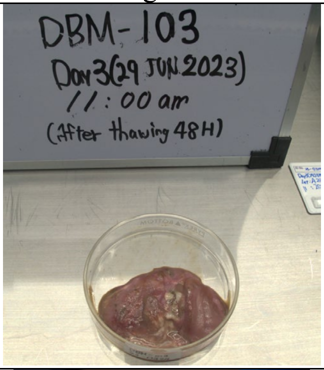	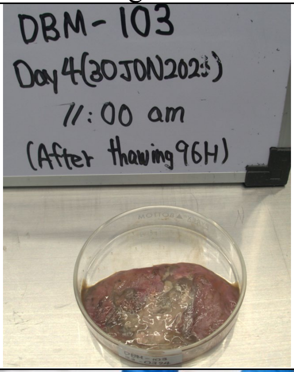
	LFD<br>(Test band intensity)	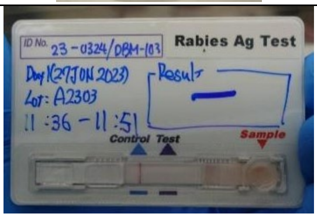<br>(0)	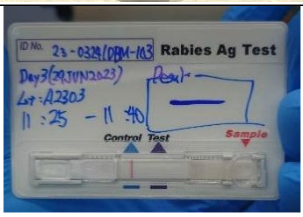<br>(0)	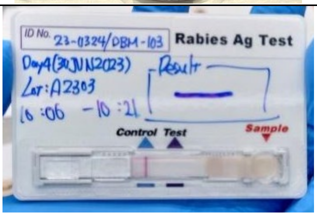<br>(0)
	DFAT	Negative	Negative	Negative

104	Brain	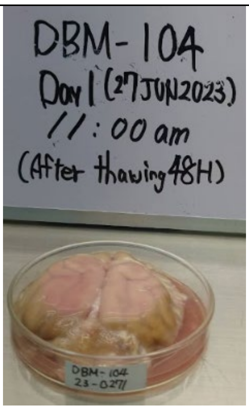	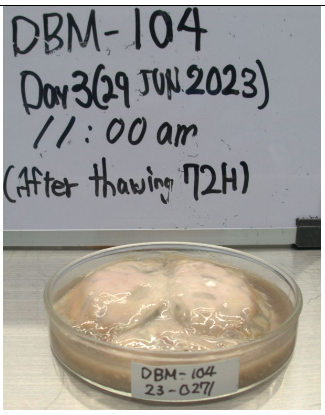	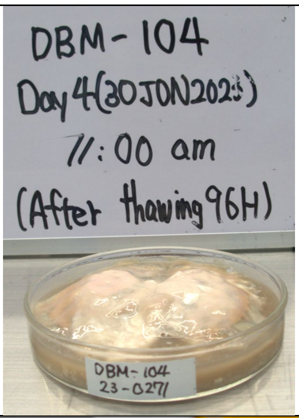
	LFD (Test band intensity)	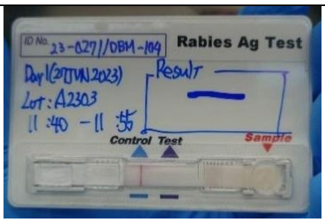 (0)	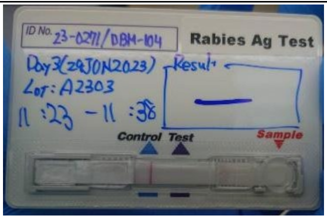 (0)	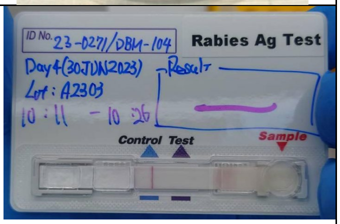 (0)
	DFAT	Negative	Negative	Negative
105	Brain	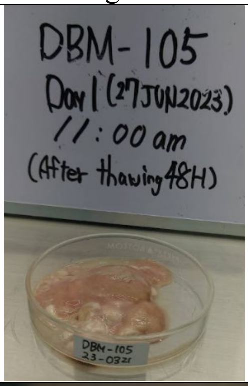	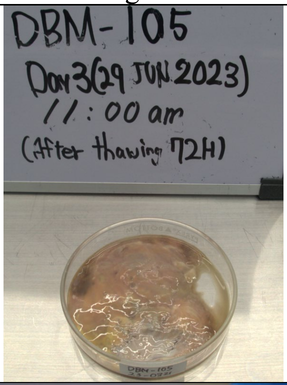	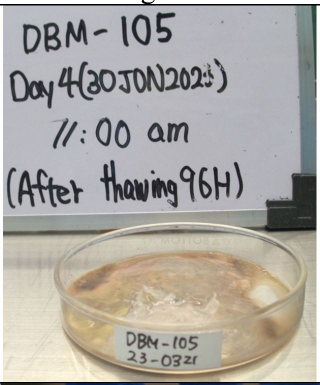
	LFD (Test band intensity)	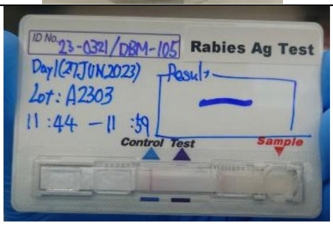 (0)	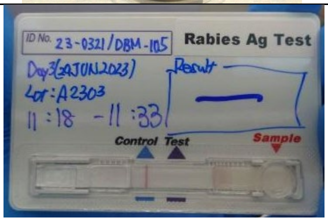 (0)	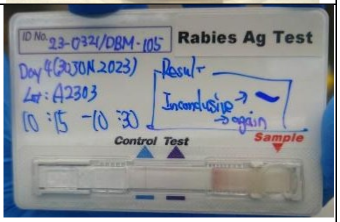 (0)
	DFAT	Negative	Negative	Negative

106	Brain	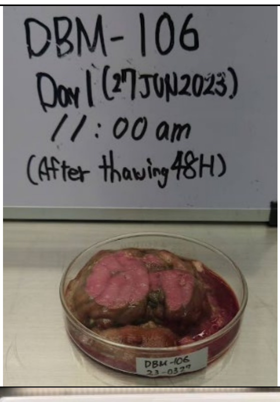	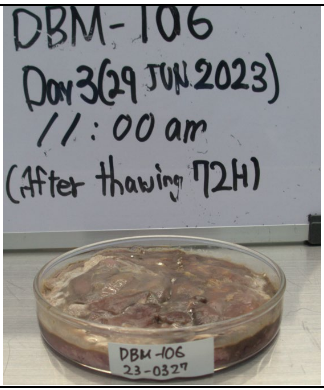	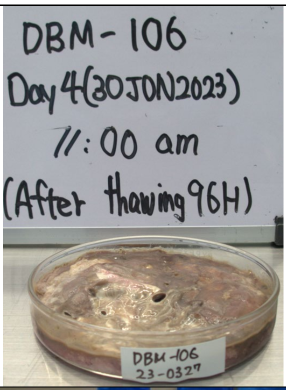
	LFD<br>(Test band intensity)	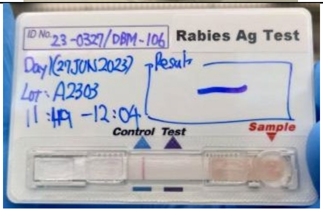<br>(0)	No image	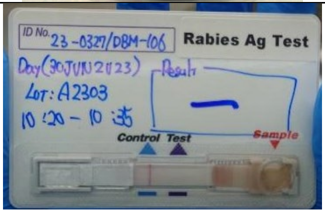<br>(0)
	DFAT	Negative	Negative	Negative
107	Brain	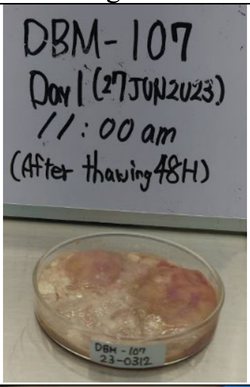	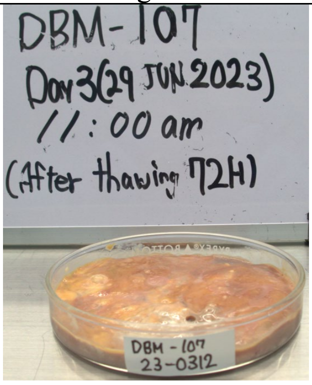	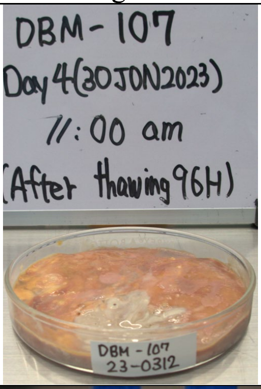
	LFD<br>(Test band intensity)	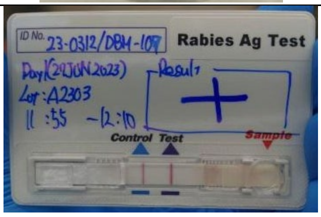<br>(2364.083)	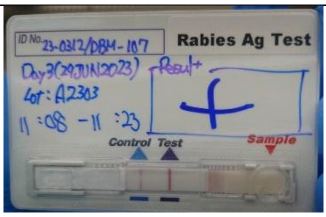<br>(2178.255)	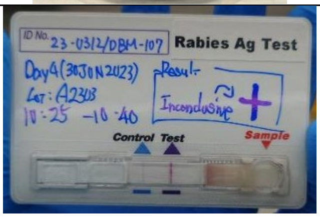<br>(1888.891)
	DFAT	Positive	Positive	Negative

<table>
<tr><td>108</td><td>Brain</td><td>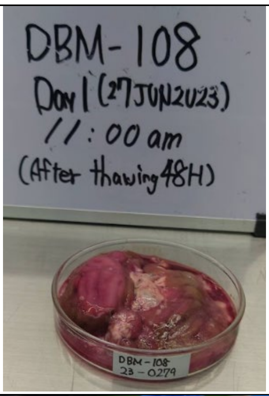</td><td>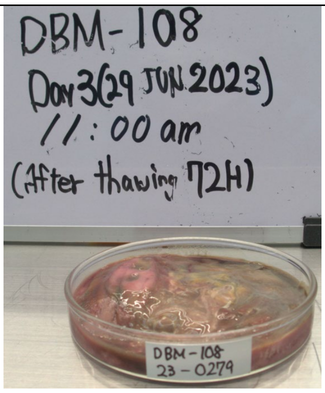</td><td>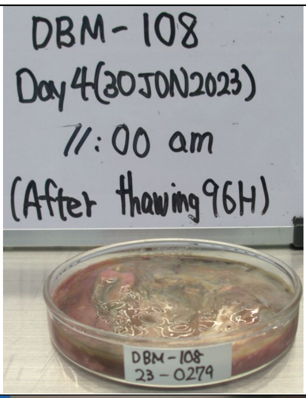</td></tr>
<tr><td></td><td>LFD
(Test band intensity)</td><td>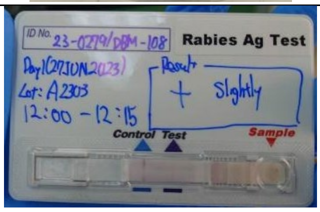
(62.121)</td><td>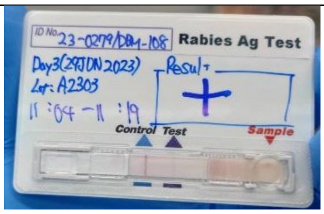
(223.192)</td><td>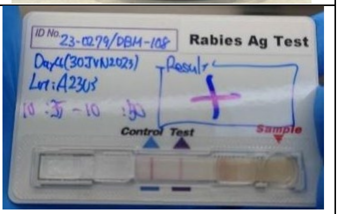
(774.991)</td></tr>
<tr><td></td><td>DFAT</td><td>Positive</td><td>Positive</td><td>Negative</td></tr>
<tr><td>109</td><td>Brain</td><td>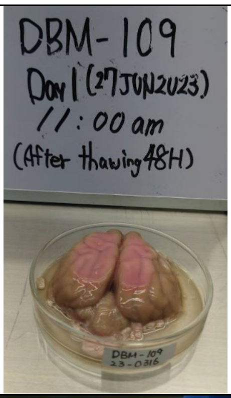</td><td>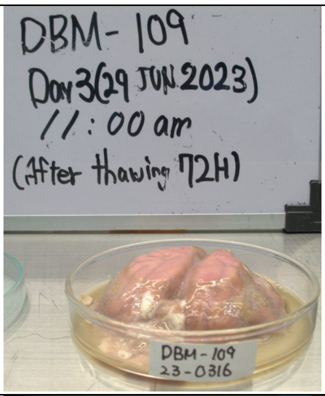</td><td>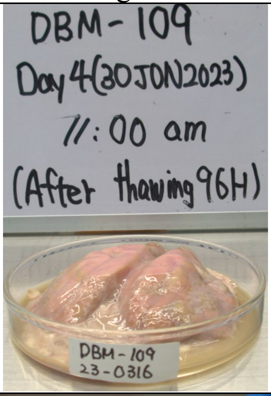</td></tr>
<tr><td></td><td>LFD
(Test band intensity)</td><td>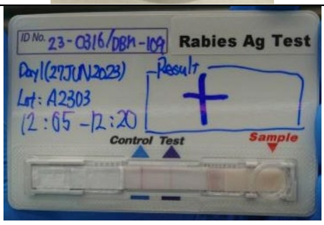
(222.435)</td><td>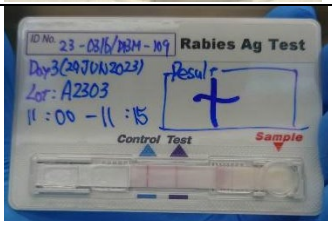
(352.385)</td><td>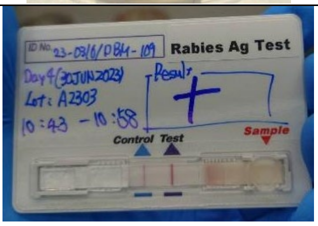
(1997.012)</td></tr>
<tr><td></td><td>DFAT</td><td>Positive</td><td>Positive</td><td>Positive</td></tr>
</table>

110	Brain	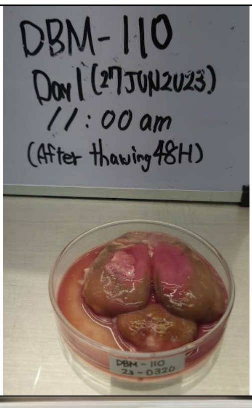	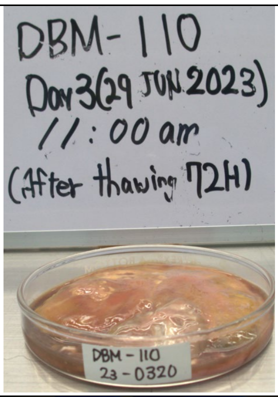	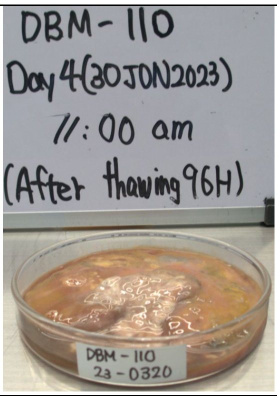
	LFD<br>(Test band intensity)	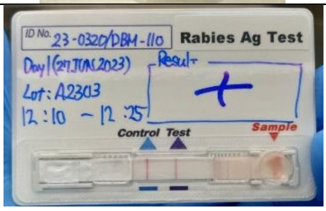<br>(492.04)	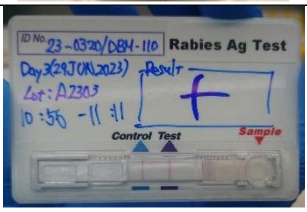<br>(372.21)	No Image
	DFAT	Positive	Positive	Positive
111	Brain	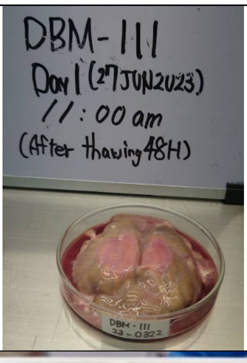	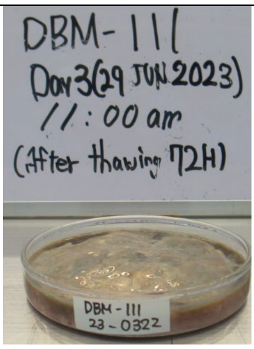	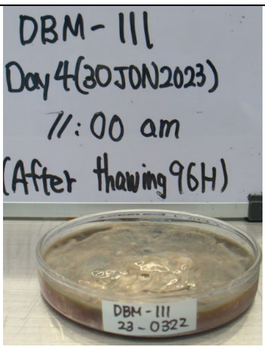
	LFD<br>(Test band intensity)	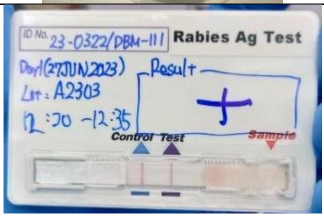<br>(623.163)	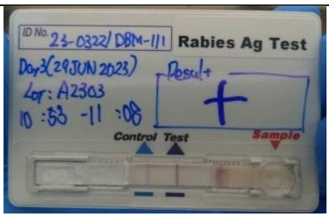<br>(670.87)	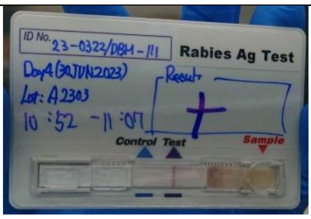<br>(668.113)
	DFAT	Positive	Negative	Negative

112	Brain	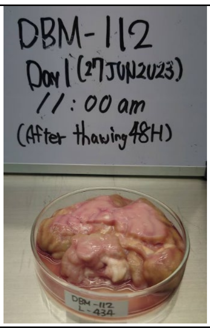	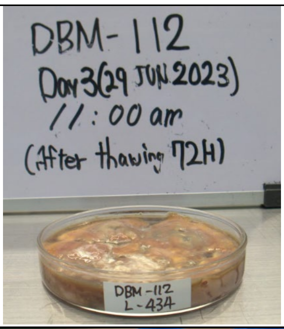	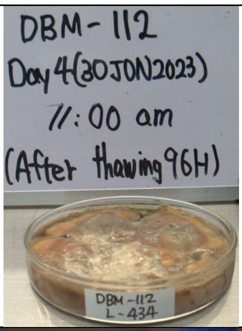
	LFD (Test band intensity)	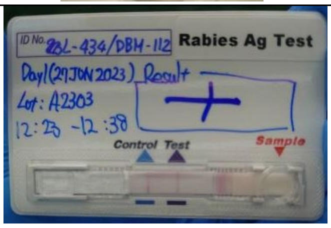 (252.44)	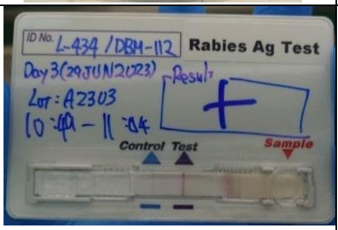 (686.11)	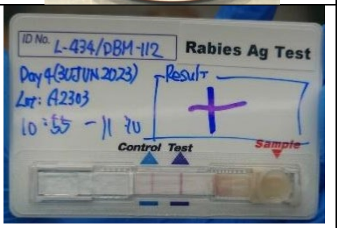 (1050)
	DFAT	Positive	Positive	Positive
201	Brain	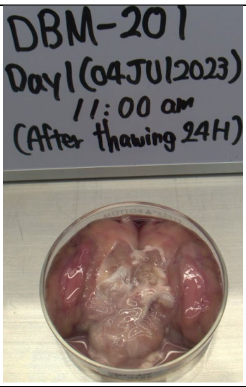	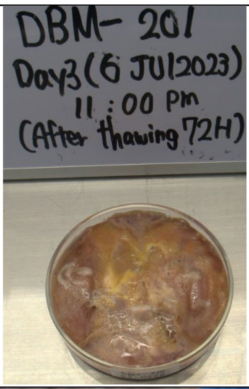	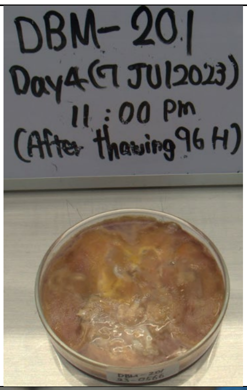
	LFD (Test band intensity)	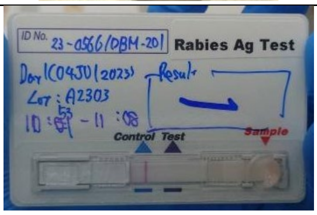 (0)	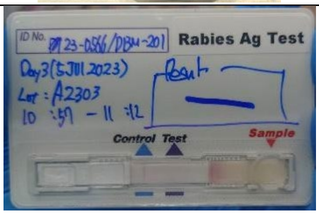 (0)	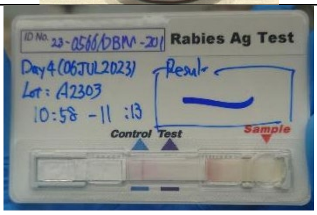 (0)
	DFAT	Negative	Negative	Negative

202	Brain	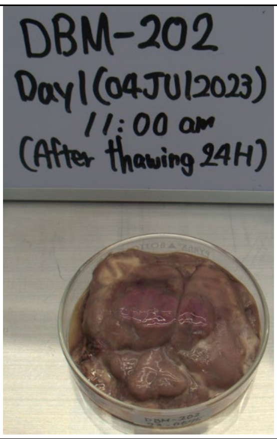	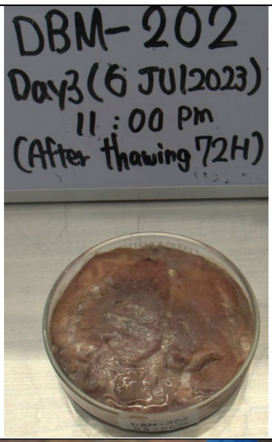	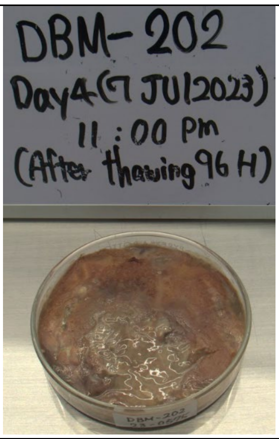
	LFD<br>(Test band intensity)	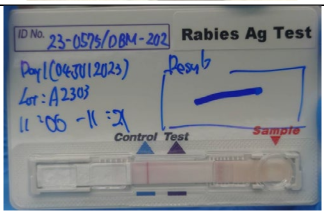 (0)	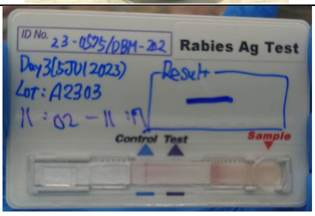 (0)	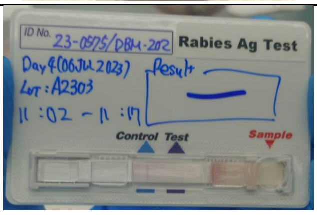 (0)
	DFAT	Negative	Negative	Negative
203	Brain	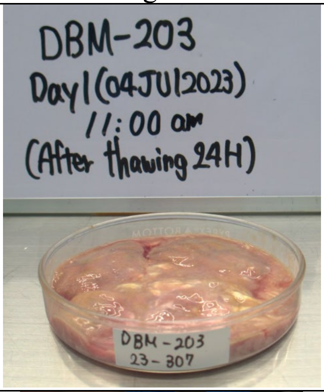	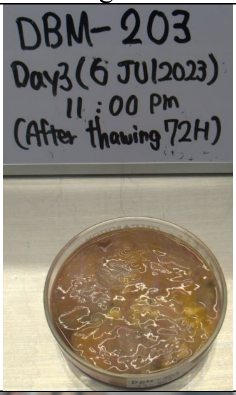	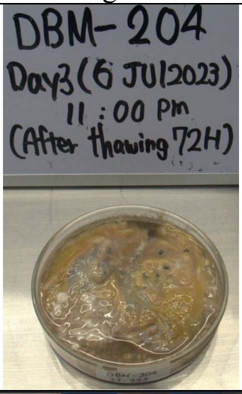
	LFD<br>(Test band intensity)	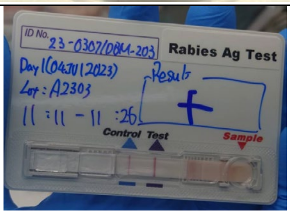 (818.99)	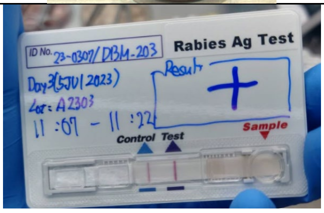 (1940.3)	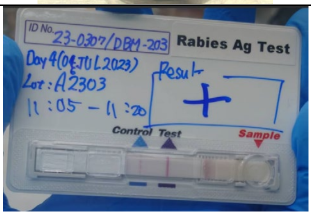 (956.23)
	DFAT	Positive	Positive	Negative

<table>
<tr><td>204</td><td>Brain</td><td>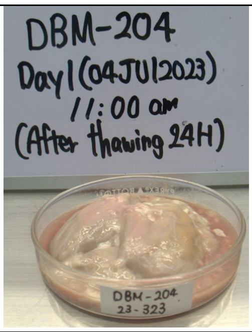</td><td>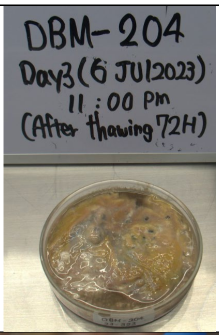</td><td>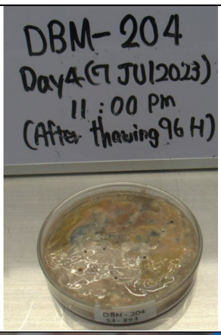</td></tr>
<tr><td></td><td>LFD
(Test band intensity)</td><td>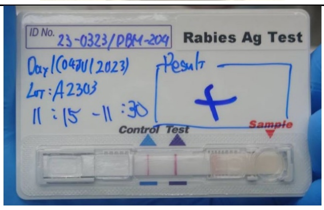(951.577)</td><td>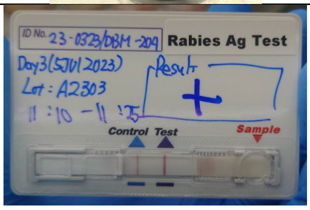(1024.577)</td><td>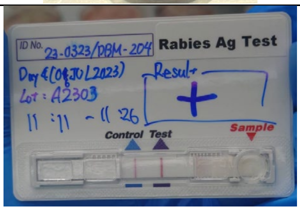(1031.577)</td></tr>
<tr><td></td><td>DFAT</td><td>Positive</td><td>Negative</td><td>Negative</td></tr>
<tr><td>205</td><td>Brain</td><td>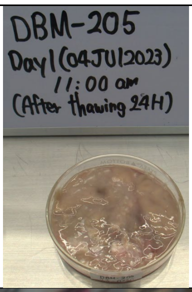</td><td>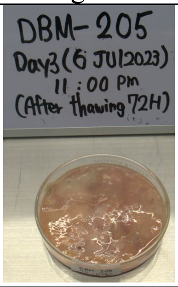</td><td>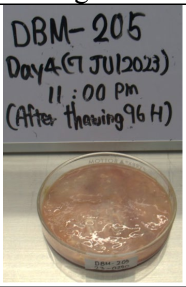</td></tr>
<tr><td></td><td>LFD
(Test band intensity)</td><td>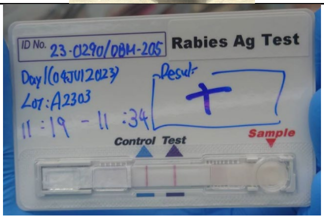(730.284)</td><td>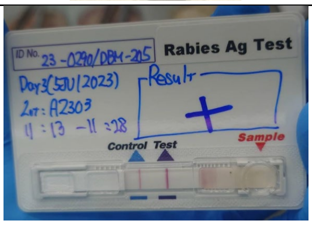(1364.698)</td><td>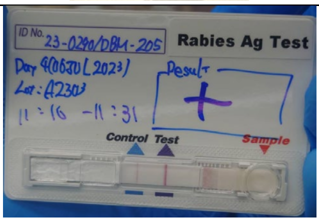(1072.698)</td></tr>
<tr><td></td><td>DFAT</td><td>Positive</td><td>Positive</td><td>Positive</td></tr>
</table>

206	Brain	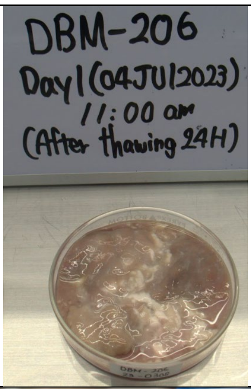	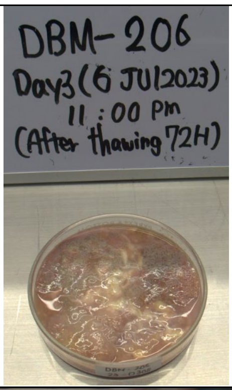	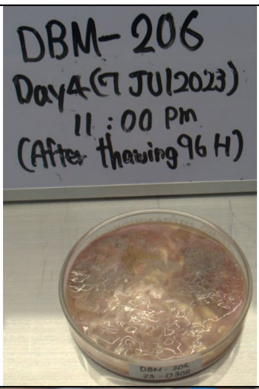
	LFD<br>(Test band intensity)	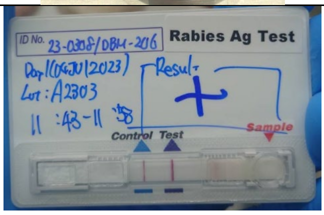<br>(1590.82)	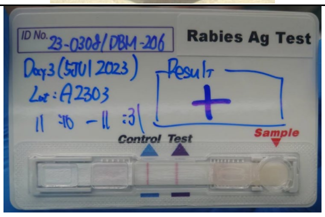<br>(714.406)	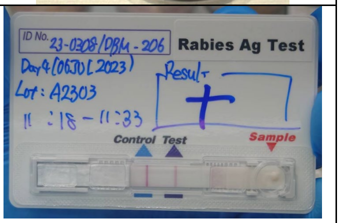<br>(551.749)
	DFAT	Positive	Positive	Positive
207	Brain	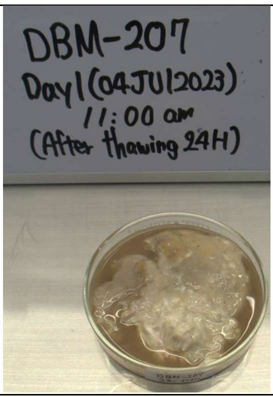	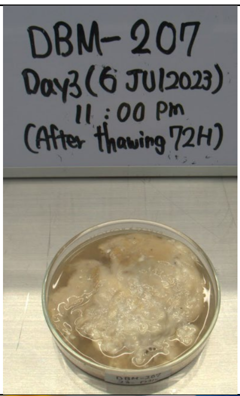	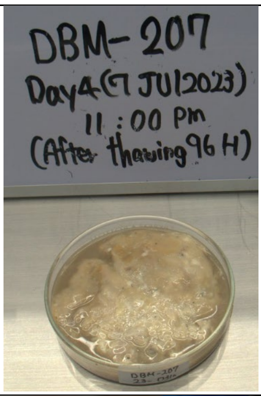
	LFD<br>(Test band intensity)	No Image	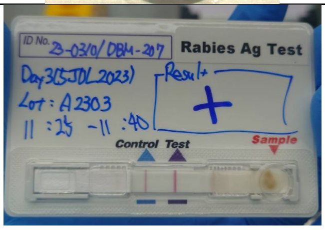<br>(716.87)	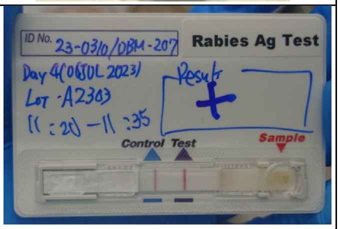<br>(773.46)
	DFAT	Negative	Negative	Negative

<table>
<tr><td rowspan="3">208</td><td>Brain</td><td>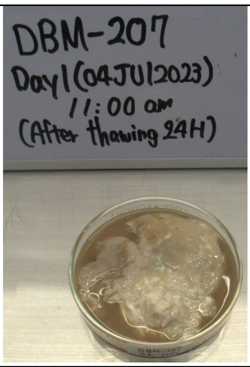</td><td>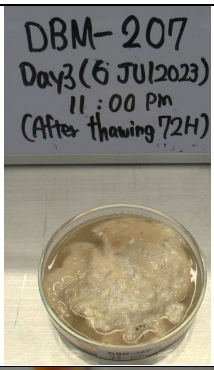</td><td>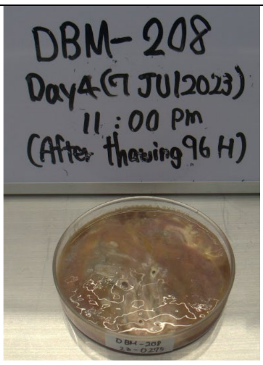</td></tr>
<tr><td>LFD
(Test band intensity)</td><td>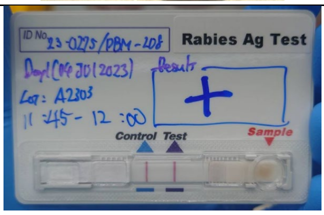(1184)</td><td>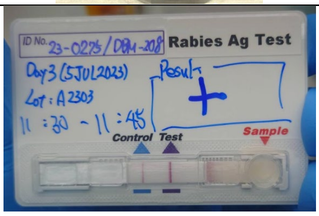(1278.2)</td><td>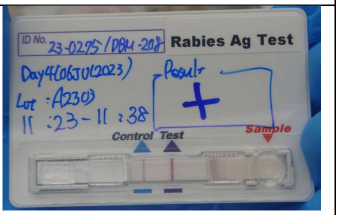(1439.4)</td></tr>
<tr><td>DFAT</td><td>Positive</td><td>Positive</td><td>Positive</td></tr>
<tr><td rowspan="3">209</td><td>Brain</td><td>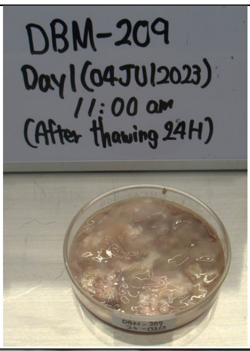</td><td>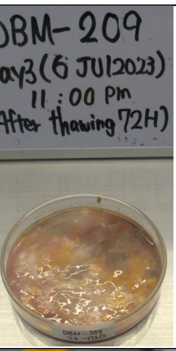</td><td>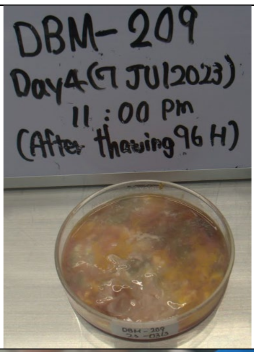</td></tr>
<tr><td>LFD
(Test band intensity)</td><td>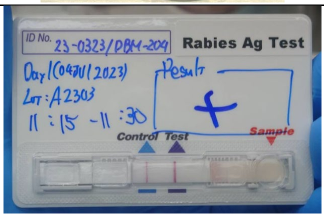(1620.4)</td><td>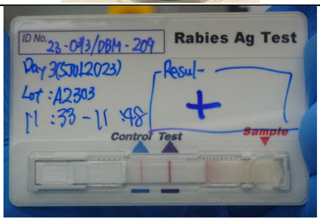(2313.8)</td><td>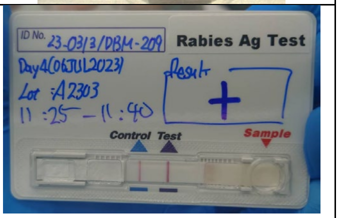(2269.8)</td></tr>
<tr><td>DFAT</td><td>Positive</td><td>Positive</td><td>Positive</td></tr>
</table>

210	Brain	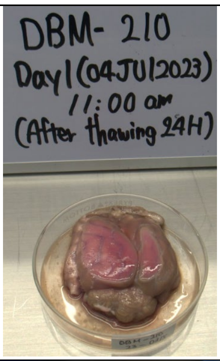	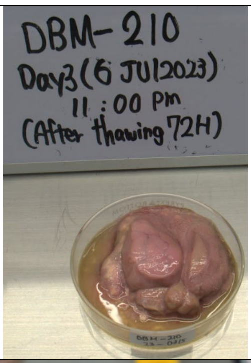	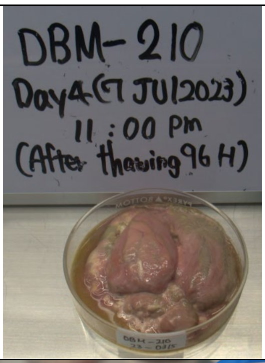
	LFD (Test band intensity)	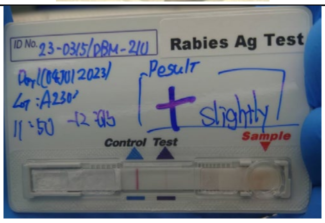 (118.778)	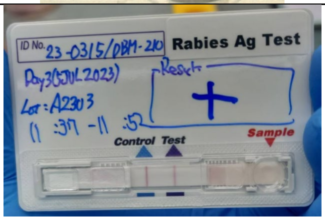 (436.92)	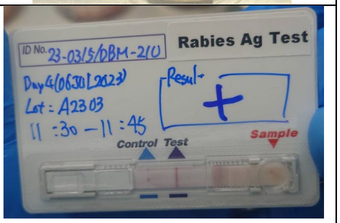 (695.406)
	DFAT	Positive	Positive	Positive
211	Brain	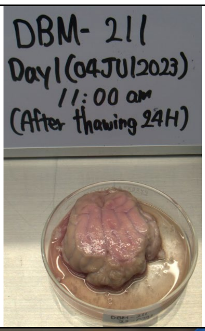	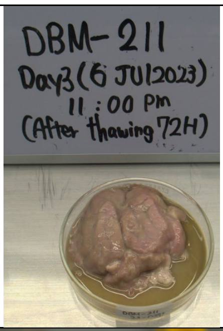	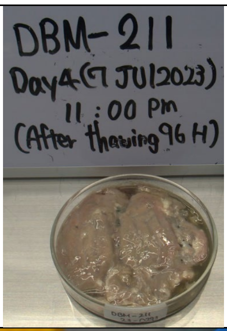
	LFD (Test band intensity)	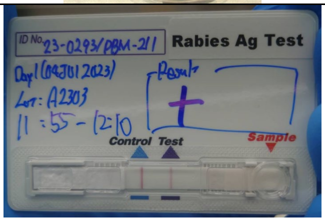 (564.75)	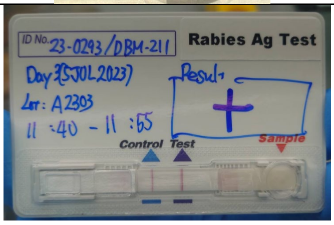 (1272.8)	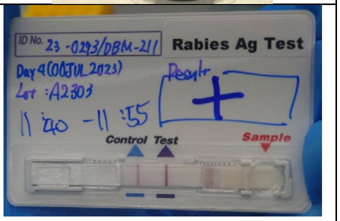 (2473.5)
	DFAT	Positive	Positive	Negative

<table>
<tr><td rowspan="3">212</td><td>Brain</td><td>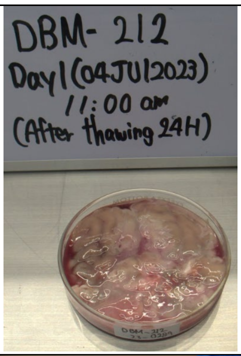
</td><td>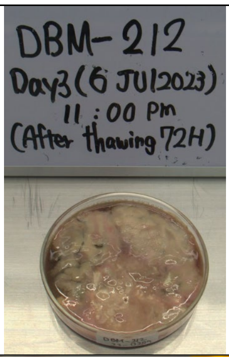
</td><td>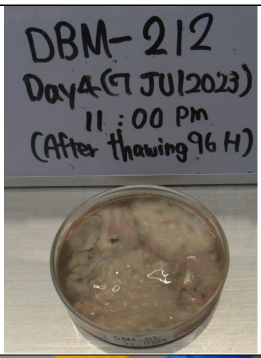
</td></tr>
<tr><td>LFD
(Test band intensity)</td><td>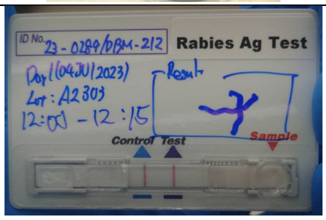

(1032.406)</td><td>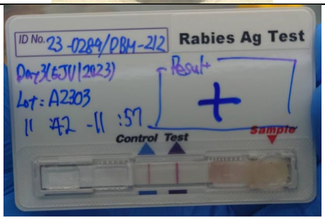

(1784.598)</td><td>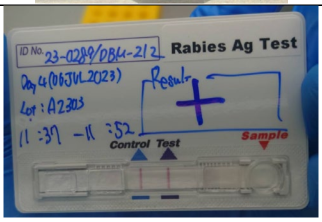

(1469.305)</td></tr>
<tr><td>DFAT</td><td>Positive</td><td>Negative</td><td>Negative</td></tr>
</table>
