# Supplementary material for: Evaluation of lateral flow devices for rabies diagnosis in decomposed animal brain samples
Source: Trop Med Health. 2025 Feb 25;53:30. doi: 10.1186/s41182-025-00699-4 (PMC11853130; doi:10.1186/s41182-025-00699-4)
Supplement: Supplementary file 4 — Additional file 4. [file 41182_2025_699_MOESM4_ESM.pdf]

Additional file 4 Table S4

Supplementary data on control and test band intensities during decomposition in DFAT-positive samples corresponding to Figure 3 (n=24)

	Test band intensity<br>Median<br>(IQR)	Control band intensity<br>Median<br>(IQR)	T/C ratio<br>Median<br>(IQR)
Day 1	651.9<br>(219.4–999.4)	1001.1<br>(588.0–1231.9)	0.76<br>(0.22–1.11)
Day 3	1321.5<br>(869.5–2117.5)	789.9<br>(421.4–1047.2)	2.06<br>(1.43–3.12)
Day 4	1454.4<br>(993.9–2009.0)	499.4<br>(220.4–837.9)	3.57<br>(2.16–6.19)

Samples with no positive bands detected or for which images were unavailable (ID=110, 207) were excluded from the analysis. The band intensity is represented by the area under the curve (AUC), calculated using the software ImageJ [https://imagej.net/ij/index.html].
IQR, Interquartile Range; T/C, Test band intensity / control band intensity
